# Assessment of Carcinogenic and non-carcinogenic risk indices of heavy metal exposure in different age groups using Monte Carlo Simulation Approach

**DOI:** 10.1038/s41598-024-81109-3

**Published:** 2024-12-05

**Authors:** B Raksha Shetty, B Jagadeesha Pai, S A Salmataj, Nithesh Naik

**Affiliations:** 1 Department of Civil Engineering, Manipal Institute of Technology, Manipal Academy of Higher Education, Manipal, 576104 India; 2 Department of Biotechnology, Manipal Institute of Technology, Manipal Academy of Higher Education, Manipal, 576104 India; 3 Department of Mechanical and Industrial Engineering, Manipal Institute of Technology, Manipal Academy of Higher Education, Manipal, 576104 India

**Keywords:** Heavy metal, Soil, Health risk indices, Monte Carlo simulation, Geospatial analysis, Civil engineering, Environmental sciences, Geochemistry

## Abstract

Dermal contact, ingestion and inhalation of heavy metal poses significant health risk in human subjects. The exposure could be via potable water, soil or air. The current experiment design focuses on soil media and exposure. Advanced probabilistic and geospatial methods are used in this study which evaluates contamination levels and health risks associated with iron (Fe), arsenic (As), zinc (Zn), copper (Cu), nickel (Ni), chromium (Cr), lead (Pb), and cadmium (Cd) in soil samples. The samples were collected and analysed using ICP-OES after tri-acid digestion, and indices such as Geoaccumulation Index (Igeo), Contamination Factor (CF), Pollution Load Index (PLI), Hazard Quotient (HQ), Hazard Index (HI), and Carcinogenic Risk (CR) were used to assess environmental impacts and health risks across the age groups via oral ingestion, inhalation and dermal contact. The current study indicates heavy metal concentrations follow the order Ni > Zn > Pb > Cu > Cr > As > Cd, with more than 60% of samples demonstrating significant pollution levels. The computational method used in the study revealed substantial non carcinogenic risk (HQ > 1) and carcinogenic risk (33%) in the population related to As exposure. Geospatial analysis and Monte Carlo simulations helped in identifying the hotspots in the tropical coastal area emphasizing need for targeted remediation focusing on As and Pb.

**Supplementary Information:**

The online version contains supplementary material available at 10.1038/s41598-024-81109-3.

## Introduction

Soil is a vital resource for human sustenance and food production, playing a fundamental role in food production and ecological balance. However, in developing countries, rapid urbanisation, rapid industrial growth, and extensive agricultural practices have led to significant environmental pollution, causing global concern^1–3^. Anthropogenic activities such as industrial emissions, mining operations, improper waste disposal, and the excessive use of pesticides and fertilizers have introduced a wide range of pollutants into the environment, with heavy metals (HM) being the most hazardous.

Arsenic (As), chromium (Cr), cadmium (Cd), lead (Pb), and nickel (Ni) are introduced into the environment and infiltrate into the soil, exposing humans to them through direct ingestion of contaminated food, inhalation of dust, and dermal absorption. The effects of these toxic metals can be acute or chronic, depending on the exposure pathway, frequency, and dosage. Vulnerable populations, including children, pregnant women, and communities living near industrial areas, are at a high risk and require special attention in public health policies and interventions.

Studies show metal exposure lead to severe health consequences. Acute exposure can cause nausea, vomiting, and abdominal issues, whereas chronic exposure can result in neurological disorders, cardiovascular issues, developmental problems, organ failure and cancer^4^. Addressing heavy metal contamination in soil requires comprehensive strategies, including environmental monitoring, pollution control, and public health education to mitigate these risks and protect human health. Given the significant reliance on soil for agriculture, food production, and livelihoods, the health hazards caused by metalloid pollution necessitate a comprehensive risk assessment. While many studies have examined heavy metal contamination in soils, few have focused on integrating health risk assessments across multiple exposure pathways using advanced computational techniques^5,6^. Reports suggest only few studies have focused on integrating health. Systematic and detailed health risk assessments are less and if present they are only for few locations. Aim of this study is to use advanced probabilistic and geospatial methods to quantify the risk associated with heavy metal contamination in the soil. Several indices and methods have been used to quantify the risks associated with heavy metal contamination in soils. Heavy Metal Pollution Index (HPI), Contamination Factor (CF), and Pollution Load Index (PLI) are the indices considered for soil quality assessment. Traditional risk assessments often entail comparing metal concentrations with the acceptable limits set by regulations. However, this strategy may not completely address the exposure uncertainties^7–11^.

The Monte Carlo simulation is a powerful statistical method used to model and analyse complex systems by generating random samples to estimate the probability of different outcomes. Monte Carlo models consider the unpredictability and variability of exposure and toxicity data when assessing health risk^12–17^. This approach allows researchers to observe a range of possible scenarios and to gain more insight into the prospective health effects associated with environmental pollutants. The accuracy of simulations can be increased by improved data collection and the use of real-time measurements^18–23^.

Earlier report suggests heavy metal contamination in peri-urban aquifers, with studies highlighting elevated levels of Ni and Cd, largely attributed to agricultural runoff and industrial activities in the selected study area. While these efforts have provided important insights into groundwater pollution, detailed health risk assessments related to soil exposure have not been explored. Geo-statistical analysis have also been conducted, to identify Pb and As near industrial zones; however, the exploration of health risks across multiple exposure pathways remains limited. In another study, contamination hotspots of Cr and Cu in agricultural soils have been mapped, offering a baseline for contamination patterns, though without the use of advanced probabilistic risk assessments or a focus on exposure variability^5,6,24,25^.

This study introduces a novel approach by combining environmental and health risk indices with Monte Carlo simulations to address targeted remediation in the selected study area. It will also explore uncertainties in exposure and toxicity data obtained. These indices were integrated with machine learning techniques using the R software. The objectives of this study were: (i) to identify heavy metal contamination hotspots using the inverse distance weighting (IDW) method and (ii) to apply integrative approaches such as environmental and human health risk indices with machine learning techniques, including Monte Carlo Simulation, to evaluate carcinogenic and non-carcinogenic health risks in different age groups. This method aims to significantly improve the assessment of heavy metal pollution and health risks in the context of soil contamination and its exposure.

This method offers a valuable tool and insight for researchers as well as policymaker in areas experiencing significant heavy contamination due to anthropogenic activities. By enhancing the understanding of the health risks posed by soil contamination, this study provides actionable insights for better environmental and public health management.

## Materials and methods

### Study area

**Fig. 1:**
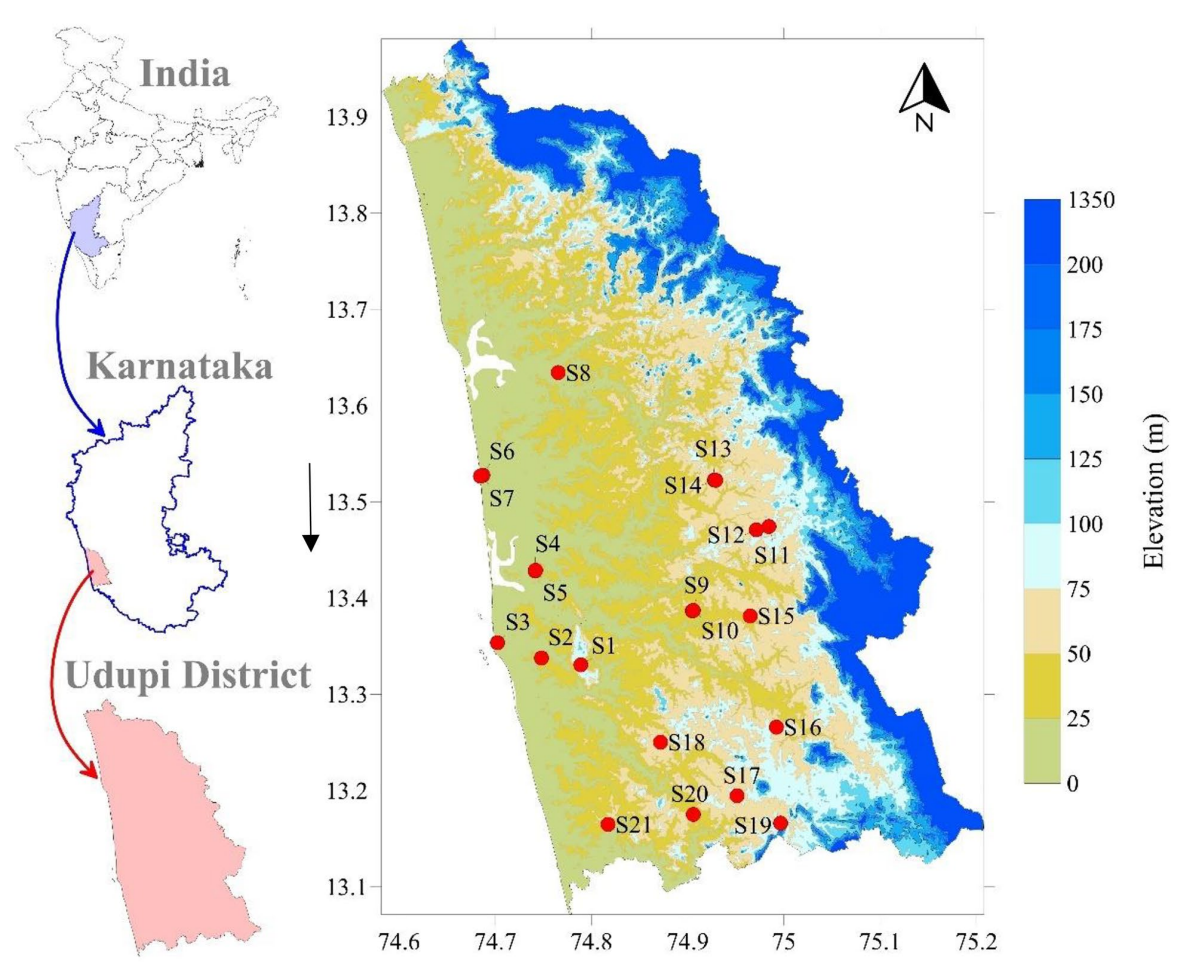
Sample locations.

The study was conducted in Udupi District, Karnataka, India. The Udupi district is a significant administrative division located in the southwestern state of Karnataka, India. The study area is positioned at a latitude of approximately 13.33° N and a longitude of 74.74° E and is situated along the coastal region of the Arabian Sea (Fig. 1). The study area houses various types of industries, such as chemical, enamel and paint, fishmeal manufacturing Units and Fish net manufacturing units, industries manufacturing clay tiles, rice mills, chemicals, thermal power plants, foundries, fish processing, and meal units. According to the 2011 Census of India, the area has a population of approximately 1,177,361. The population density was approximately 329 individuals per sq.km. The majority of people living in the area are under 35 years of age, making it a young demographic. Nearly 10% of people are younger than six years old, while 11% are older than 60 years.

The population is predominantly rural, with approximately 70% residing in rural areas and 30% residing in urban areas. Public health centres are located across various parts of the rural and semi-urban areas of the district, which provide essential healthcare services.

### Sample collection and analysis

**Fig. 2:**
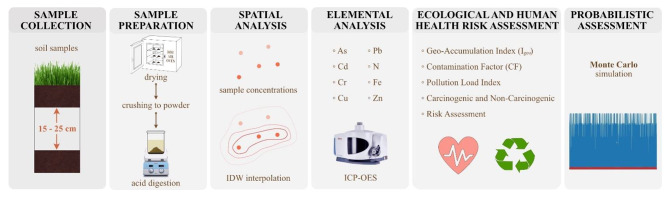
Illustration of the step-by-step methodology employed in the study.

To assess the heavy metal contamination in the soil and their associated environmental and human health risk, soil samples were collected from 21 locations (Fig. 1). The sites selection was based on high anthropogenic activity which leads to release of contaminants. The samples were collected were near industrial areas such as chemical, enamel and paint, fishmeal manufacturing units and fish net manufacturing units, industries manufacturing clay tiles, rice mills, chemicals, thermal power plants, foundries, fish processing, and meal units and their proximity to potential contamination sources. These were chosen based on the likelihood of human exposure to contaminants, particularly in communities located near these industrial zones. Uniformity in the site selection criteria ensured the representativeness and reliability of the sample. At each sampling location, three subsamples were collected as controls. The samples were collected during the dry and wet seasons from 2022 to 2023. The surface was cleared with a wooden spatula and the soil was dug to a depth of 15–25 cm. The samples were then collected via thorough mixing. The geographical coordinates of each point were accurately recorded using a GPS device. The soil samples were dried at 60 °C and then subjected to the triacid (HNO3 + HCL + HF) digestion technique. Contaminants were quantified using inductively coupled plasma-optical emission spectroscopy (ICP-OES) at the Environmental Research Laboratory at the Manipal Institute of Technology, Manipal, India. This is a well-established instrument that can detect contaminant concentrations of up to 10ug/l. To ensure accuracy and precision, necessary quality control measures were tested for metals and metalloids such as As, Hg, Cd, Ni, Fe, Pb, Cr, Al, Cu, Zn. These concentrations were measured and recorded in mg/kg. The methodology followed in from sample collection to assessment is given in Fig. 2.

#### Quality control

Blanks were prepared with each batch of sample digestion to ensure to trace the source of error during the elemental analysis. An instrumental blank was added during analysis after every 10 to avoid interference of residue. Standards were prepared for 0.01 mg/L, 0.05 mg/L, 0.1 mg/L, 0.5 mg/L, 1 mg/L, 2.5 mg/L, 7.5 mg/L, 10 mg/L, 15 mg/L, 20 mg/L, 25 mg/L and 50 mg/L. Samples with concentration of elements greater than 50 mg/L were re-diluted and assessed. For each element, R^2^ > 0.98 was maintained to ensure linearity and reproducibility. Accuracy and precision of the analysis is assessed using duplicate samples and certified reference materials (BCR^®^-667). RSD was less than 5%. Triplicate measurements were carried out during the analysis for precision.

### Spatial analysis

Heavy metal concentrations from the collected samples were georeferenced, and the values of each heavy metal in the unsampled location were evaluated based on the assumption that point data that are closer to each other are more similar than those that are further away. QGIS 3.18 version was used for spatial prediction using the inverse distance weighted method.

#### Accuracy of spatial prediction

The accuracy of the model was measured using the root-mean-square error (RMSE). The RMSE value for this prediction was 0.0025, indicating a better fit of the model to the data.

### Pollution Assessment

**Environmental risk assessment in soil**.

#### Geo-accumulation index (Igeo)

Igeo approach was used to quantify the concentration of metal accumulation in the sediment over baseline levels. This index provides a gauge of the intensity of pollution by comparing current metal concentrations with baseline or pre-industrial levels^26–29^. Table 1 provides the Igeo, PLI and CF status classification. The Geo-accumulation Index for soil was calculated using Eq. 1 to identify the level of metal pollution and its environmental impact.

1 $$Igeo = \:lo{g_2}\frac{{Cx}}{{1.5X\:Bx}}$$

Where Cx refers to the concentration of the element x in soil sample (mg/kg) and Bx is the geochemical background value of the element x. 1.5 is a constant used to account for natural variations in background concentrations. Bx values for each element assed is given as follows: As = 5 mg/kg; Cd = 0.3 mg/kg; Cr = 75 mg/kg; Cu = 20 mg/kg; Fe = 50,000 mg/kg; Ni = 30 mg/kg; Pb = 20 mg/kg and, Zn = 50 mg/kg.

#### Contamination factor (CF)

CF is a commonly used metric in environmental research for evaluating the level of soil pollution caused by HMs. It is determined by the ratio of the concentration of a heavy metal in a contaminated soil sample to its concentration in a reference, background, or uncontaminated sample, which is typically from the same region^30–33^. Sediment sample contamination can be assessed using the CF, which can be calculated using Eq. 2.

2 $$CF=\:\frac{\text{C}\text{x}\:\left(\text{s}\text{e}\text{d}\text{i}\text{m}\text{e}\text{n}\text{t}\right)}{\text{C}\text{x}\:\left(\text{r}\text{e}\text{f}\text{e}\text{r}\text{e}\text{n}\text{c}\text{e}\right)}$$

where Cx(sediment) is the measured concentration of the element x in the sample (mg/kg) and Cx(reference) is the concentration of same element x (mg/kg). Cx values for each element assed is given as follows: As = 5 mg/kg; Cd = 0.3 mg/kg; Cr = 75 mg/kg; Cu = 20 mg/kg; Fe = 50,000 mg/kg; Ni = 30 mg/kg; Pb = 20 mg/kg and, Zn = 50 mg/kg.

#### Pollution load index (PLI)

PLI is a cumulative measure used to assess the overall level of heavy metal contamination in the soil. The geometric mean of the CF for each of the metals was used to compute it. The PLI values were calculated using Eq. 3.

3 $$PLI = {\left( {C{F_{As}} \times C{F_{Cd}} \times C{F_{Cr}} \times C{F_{Cu}} \times C{F_{Fe}} \times C{F_{Ni}} \times C{F_{Pb}} \times C{F_{Zn}}} \right)^{1/n}}$$

Where, CF_As_, CF_Cd_​ CF_Cr_​, CF_Cu_​, CF_Fe_​, CF_Ni_​, CF_Pb,_ CF_Zn_ are contamination factor values of As, Cd, Cr, Cu, Fe, Ni, Pb and Zn respectively. n is the number of metals analysed.

**Table 1:** Classification of Igeo, PLI, and CF values^34–36^.

PLI Value		Status
≤ 1		No metal pollution
> 1		Metal pollution exists
Igeo Value	**Class**	**Status**
> 5	6	Very strongly polluted
> 4–5	5	Strong to very strongly polluted
> 3–4	4	Strongly polluted
> 2–3	3	Moderately to strongly polluted
> 1–2	2	Moderately polluted
> 0–1	1	Unpolluted to moderate polluted
< 0	0	Practically unpolluted
CF values		**Status**
CF < 1		Low contamination
1 ≤ CF < 3		Moderate contamination
3 ≤ CF < 6		Considerable contamination
CF > 6		Very high contamination

### Human Health Risk Assessment

The EPA recommended method for health risk assessment was used in this study to evaluate the potential health effects of exposure to metal(loid)s. Human health risks from contaminants are categorized into two types: cancer-causing and non-cancerous hazards. While carcinogenic risk (CR) assessments are conducted, especially for carcinogens, non-carcinogenic risk assessments (non-CR) can be applied to both carcinogenic and non-carcinogenic contaminants. To achieve this, the concentration of each contaminant was first determined, followed by qualitative and quantitative estimations of the associated health risks. Primary toxic heavy metals were evaluated for their potential to cause health problems, namely As, Pb, Fe, Mn, Pb, Ni, and Cd. Human health risks are assessed based on exposure routes, specifically oral ingestion, dermal contact and inhalation of polluted media. Health risks from these routes were evaluated in adults (18–40 years), teenagers (12–18 years), and children (2–12 years). An HQ or HI value greater than one adds to the health risk associated with non-cancer risk. When the CR of the carcinogen is less than 1 × 10^− 6^, the risk of developing cancer is minor and can be disregarded. However, cancer may result in CR values > 1 × 10^− 4^. Additionally, cancer risk was low when CR values < 1 × 10^− 6^^37^.

#### Exposure assessment models

Models of exposure assessment are helpful, as they offer a methodical way to calculate the amount, frequency, and length of time that people are exposed to toxins through various exposure pathways. Here, soil samples were assessed for three routes of exposure: oral ingestion, dermal absorption and inhalation.

#### Ingestion exposure

Ingestion of contaminated soil is a key exposure pathway for heavy metals, particularly in children and individuals living or working near polluted areas. Soil particles can easily be ingested through hand-to-mouth activities, especially in outdoor environments. Children, in particular, can ingest substantial amounts of soil during play, while occupational or residential exposure in contaminated areas also contributes to this pathway. The health risks associated with this route are assessed by calculating the daily intake via soil ingestion using Eq. (4). The values used for these calculations are listed in Table 2.

4 $$\:{\rm{Daily}}\,{\rm{Intak}}{{\rm{e}}_{{\rm{(ingestion)}}}} = \frac{{{\rm{Cs}}\: \times \:{\rm{IR}}\:\left( {{\rm{ingestion}}} \right)\: \times \:{\rm{EF}}\: \times \:{\rm{ED}}}}{{{\rm{BW}}\: \times \:{\rm{AT}}}}\,\,\,\,\,\, \times {10^{ - 6}}$$

Where, Cs = measured concentration of heavy metal s in soil (mg/kg); IR(ingestion) = Soil Ingestion Rate (mg/day); exposure frequency (days/year); ED = exposure duration (years); BW = body weight (kg); AT = average time of exposure (days); Daily Intake_(ingestion)_ is measured in mg/kg/day.

**Table 2:** Standard values used for estimation of daily intake via ingestion^38–40^.

	Adults	Teenagers	Children
IR(ingestion)(mg/day)	100	150	200
IR (inhalation) (mg/cm^3^)	20	15	10
ED ( years)	35	15	3
EF(days/year)	365	365	365
BW (kg)	70	35	15
AT (days)	EFx365	EFx365	EFx365

#### Dermal exposure

Contaminants absorbed through the skin are referred to as dermal exposures. This pathway is particularly relevant for individuals who come into direct contact with polluted soil, water, or surfaces. In the context of HM contamination in soil, dermal exposure can occur through activities such as working in contaminated fields and playing in contaminated areas. The daily intake from dermal absorption was calculated using Eq. 5. The values used for the calculations are listed in Table 3.

5 $$\:{\rm{Daily}}\,{\rm{Intak}}{{\rm{e}}_{{\rm{(dermal)}}}}{\rm{ = }}\frac{{{\rm{Cs}}\: \times \:{\rm{SA}}\: \times \:{\rm{AF}} \times \:{\rm{ABS}}\: \times {\rm{EF}} \times \:{\rm{ED}}}}{{{\rm{BW}}\: \times \:{\rm{AT}}}}\,\,\, \times \,{10^{ - 6}}$$

Where, Cs​: measured concentration of heavy metals in soil (mg/kg); SA: Surface area of skin exposed (cm²); AF: Adherence factor (mg/cm² for soil); ABS: Dermal absorption factor; EF: Exposure frequency (days/year); ED: Exposure duration (years); BW: Body weight (kg); AT: Averaging time (days); Daily Intake_(dermal)_ is measured in mg/kg/day.

#### Inhalation exposure

An inhalation exposure model for dust generated from soil is used to assess the health risks associated with breathing in airborne particles that originate from contaminated soil. This model focuses on particulate matter that is resuspended into the air from soil due to natural factors (such as wind) or human activities (such as construction or farming). The airborne dust may contain hazardous substances like heavy metals, which can be inhaled by individuals living or working in contaminated areas. Daily consumption of HM from inhalation can be calculated using Eq. 6.

6 $${\rm{Daily Intak}}{{\rm{e}}_{{\rm{(inhalation)}}}} = \:\frac{{{\rm{Cs}} \times \:{\rm{IR}}\:\left( {{\rm{inhalation}}} \right) \times \:{\rm{EF}} \times \:{\rm{ED}}}}{{{\rm{BW}} \times \:{\rm{AT}} \times \:{\rm{PEF}}}}$$

Daily Intake _(inhalation)_ = Average daily dose of the contaminant (mg/kg/day); Cs = Concentration of the contaminant s in soil (mg/kg); IR: Inhalation rate (m³/day); EF: Exposure frequency (days/year); ED: Exposure duration (years); BW: Body weight (kg); AT: Averaging time (days); PEF: Particle emission factor (m³/kg), which converts soil concentration to airborne concentration. The values used for these parameters are given in Table 2.

**Table 3:** Standard values used for estimation of daily intake via dermal absorption^39–41^.

	Adults	Teenagers	Children
SA (cm^2^)	6600	5000	2800
AF(mg/ cm^2^)	35	15	3
ABS	365	365	365
BW (kg)	70	35	15
AT (days)	EFx365	EFx365	EFx365

**Table 4:** Reference dose values for the metals used for risk assessment^42–46^.

Metals	RfD_ing_	RfD_derm_	CSF_ing_	CSF_derm_	CSF_inh_
As	3 × 10^− 4^	1.23 × 10^− 4^	1.5	1.5	15.1
Cd	1 × 10^− 4^	1 × 10^− 5^	0.38	6.3	6.3
Fe	7 × 10-1	7 × 10-1	-	-	-
Hg	3 × 10^− 4^	2.1 × 10^− 5^	-	-	-
Ni	140 × 10^− 3^	1.8 × 10^− 3^	-	-	-
Ni	-	-	0.84	0.84	0.84
Pb	3.5 × 10^− 4^	5.25 × 10^− 4^	0.0085	0.042	-
Zn	0.3	-	-	-	-

#### Carcinogenic and non-carcinogenic risk assessment

Metals, such as As, Hg, Cd, Ni, Fe, Mn, have been identified as hazardous materials for the community. After identification, the exposure routes of each of these metals (dermal absorption and oral ingestion) were studied. Hazard quotients (HQ) and cancer risks (CR) were evaluated using reference dose and cancer slope factor values. Some metals were not included in the assessment because of the absence of reference dose and cancer slope factor values. Equation 7 was employed to determine the non-CR health risks associated with metals such as As, Pb, Zn, Ni, Mn, Ni, and Fe from oral consumption and skin absorption in adults, teenagers, and children.

7 $${\rm{H}}{{\rm{Q}}_{{\rm{oral/dermal/inhalation}}}}{\rm{ = }}\:\frac{{{\rm{DI}}\left( {{\rm{dermal}}} \right)/{\rm{DI}}\left( {{\rm{oral}}} \right)/{\rm{DI}}\left( {{\rm{inhalation}}} \right)}}{{{\rm{RfD}}\left( {{\rm{dermal}}} \right)/{\rm{RfD}}\left( {{\rm{oral}}} \right)/{\rm{RfD}}\left( {{\rm{inhalation}}} \right)}}$$

Where, DI is daily intake of HM from dermal contact, oral ingestion, and inhalation. RfD is the reference dose for each element. The values used for the calculation are given in Table 4.

The hazard Index (HI) was used to evaluate the combined effect of the health risk from each metal. This is given by Eq. 8.

8 $$HI =\:\sum\:HQ$$

Where, HI is hazard index; HQ is hazard quotient value of specific element in an exposure route.

Only carcinogens that exceeded the permissible limit were chosen for the CR assessment in this study. The metals considered for the CR assessments were As, Cd, Pb, and Ni. The carcinogenic health risks from dermal contact and oral ingestion were calculated using Eq. 9.

9 $$CR = {\rm{ }}DI{\rm{ }} \times {\rm{ }}CSF$$

where CSF is the conversion slope factor of the HMs. DI is daily intake from oral ingestion/ dermal contact or inhalation. The values used in the health risk assessment are listed in Table 4.

### Monte Carlo simulation

the results. To address these uncertainties, probabilistic models are used to provide a clearer picture of risk^47^. In this study, we applied Monte Carlo simulation to improve the reliability of the analysis^18,21,48–50^. HQ values for As, Cd, Fe, Hg, Mn, Ni, Pb, and Zn were calculated for children and adults (oral ingestion and dermal contact) as well as the CR of As, Cd, Ni, and Pb in both age groups. To perform Monte Carlo simulations and determine the probability risk associated with carcinogenic and non-carcinogenic exposure in adults, teenagers, and children, the R code was run 10,000 times in RStudio. The distribution types of the parameters used were as follows, As: Log-normal; Cd: Log-normal; Ni: Gamma; Ingestion Rate (Normal); Body Weight: Normal; Skin surface area: constant; Cancer slope factor: constant.

## Results

### Availability of heavy metals in soil

### Environmental risk assessment

**Fig. 3:**
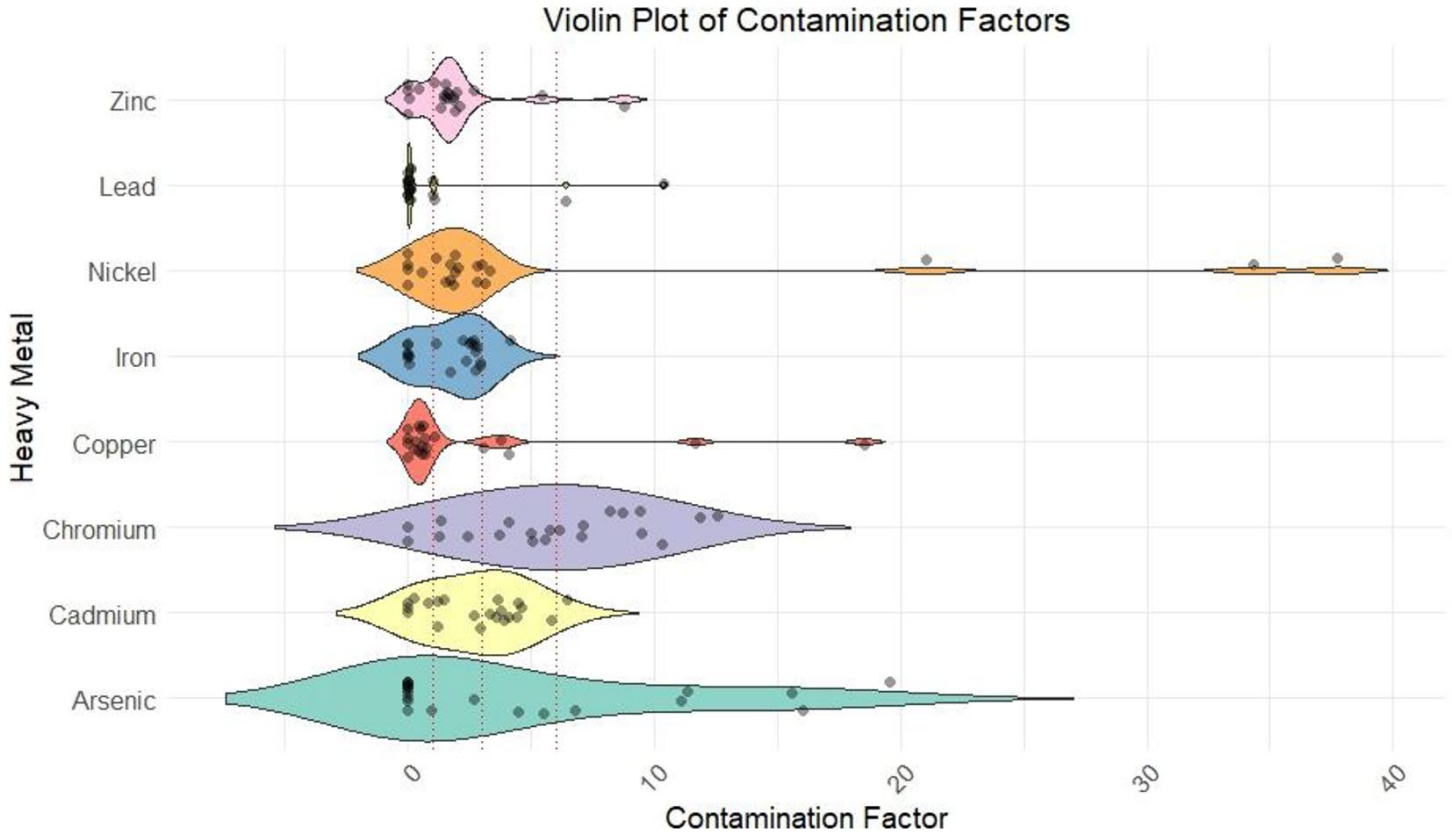
Contamination factor values of HM in soil.

**Fig. 4:**
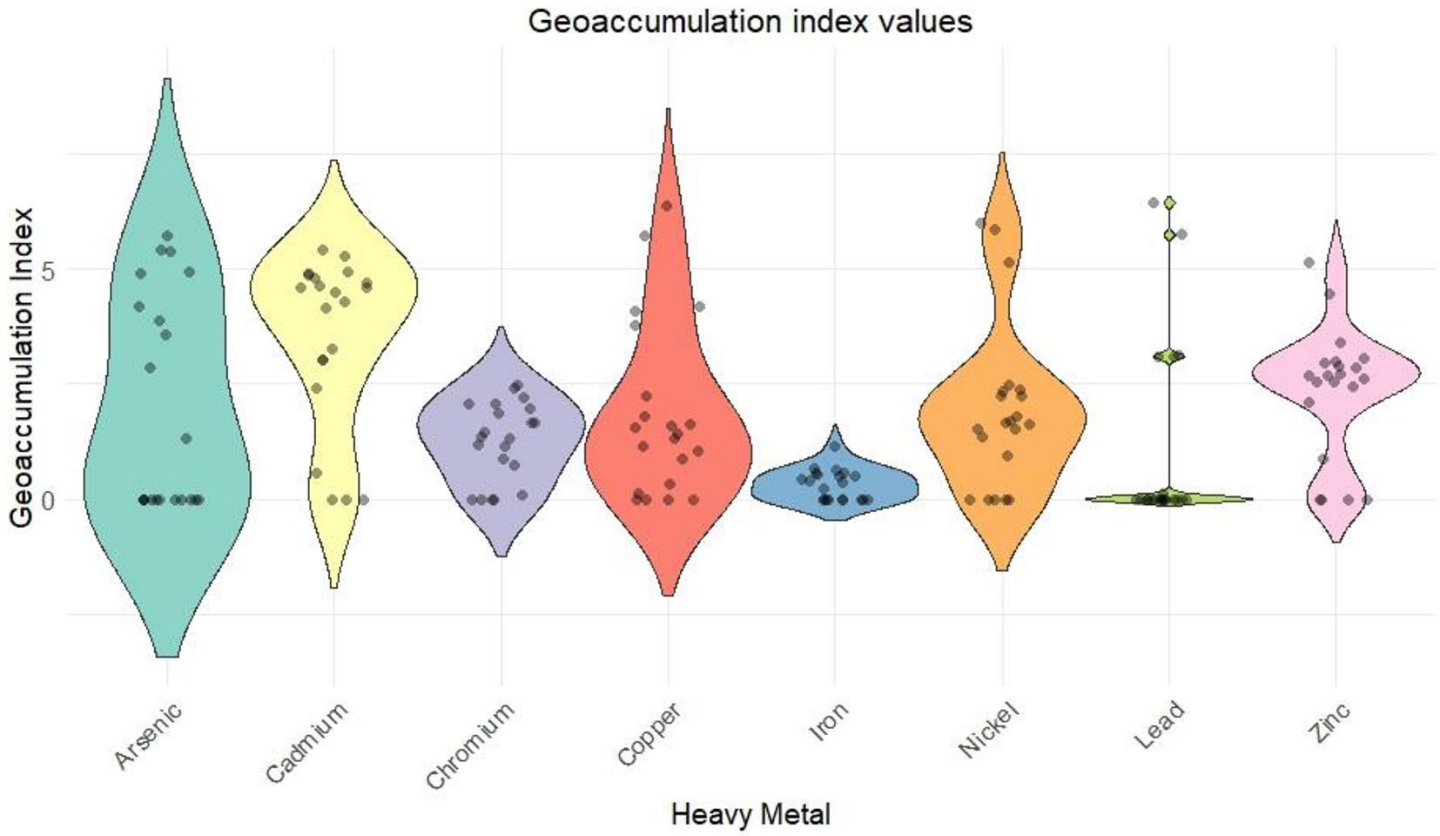
Igeo index values for soil samples.

**Fig. 5:**
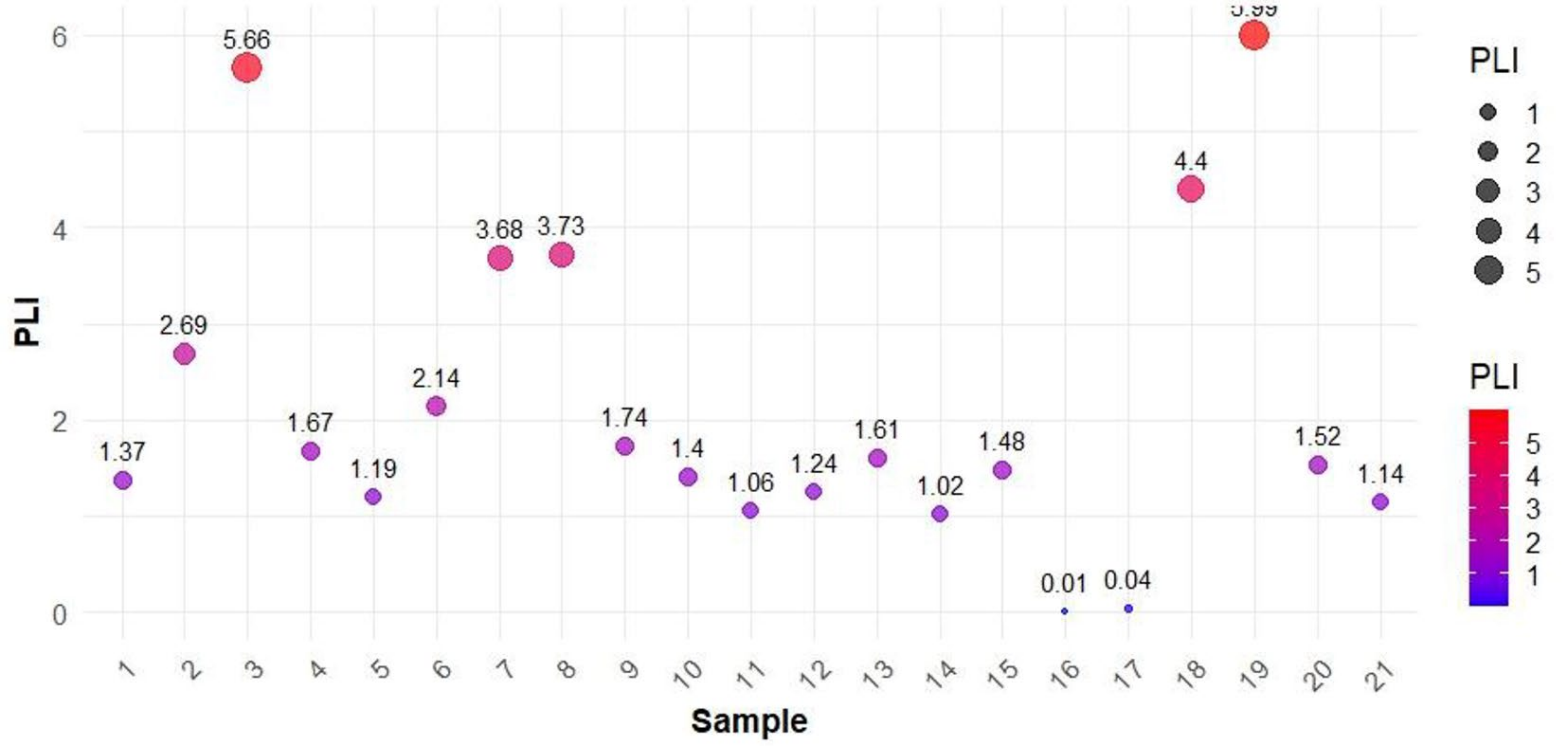
Pollution load index values of soil samples.

Figures 3, 4 and 5 shows the variation of CF values, Igeo values and PLI values of the soil samples respectively. CF changed from 1 to 20 for As, 1–6 for Cd, 2–12 for Cu, 1–4 for Fe, 1–30 for Ni, 1–9 for Pb, 1-7.5 for Zn. Metals such As, Ni, and Cu exhibited high CF values at several points, and Cd, Cr, and Fe showed moderate contamination with peaks indicating higher levels in specific samples. Pb and Zn exhibited lower CF values with occasional peaks, indicating higher contamination. Value of Igeo values for As exceeded 5 in majority of the samples indicating extreme As contamination. Where Cd, Cr, Ni, and Zn showed moderate contamination. Fe and Pb showed low contamination. PLI values of the eight HMs varied from 0.006 to 5.994 with an average value of 2.132. Overall, 91.3% of samples were contaminated with heavy metals.

### Health risk assessment

#### Non-carcinogenic risk from oral ingestion

**Fig. 6:**
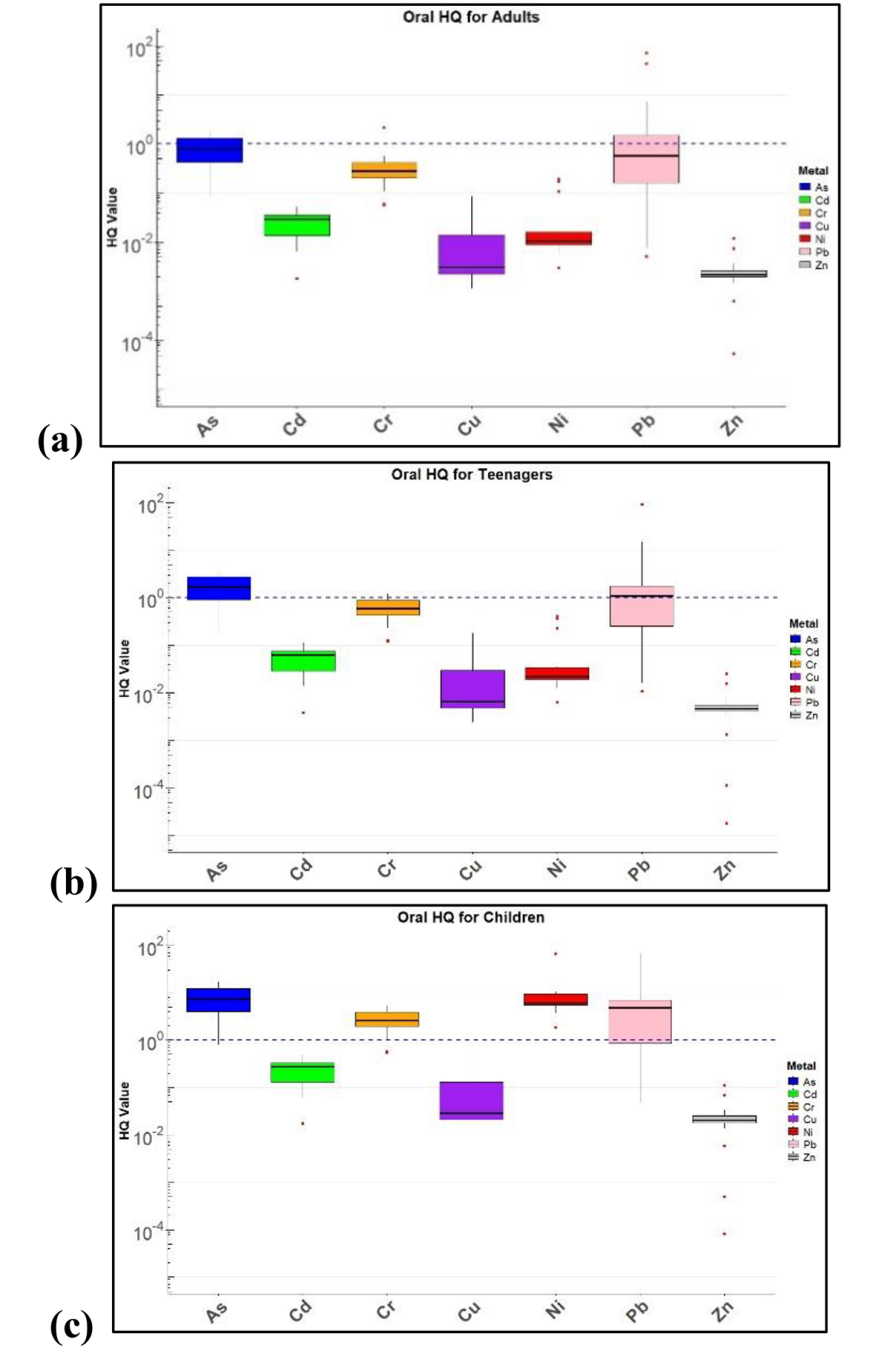
HQ values for oral ingestion in (**a**) Adults, (**b**) Teenagers and (**c**) Children.

**Fig. 7:**
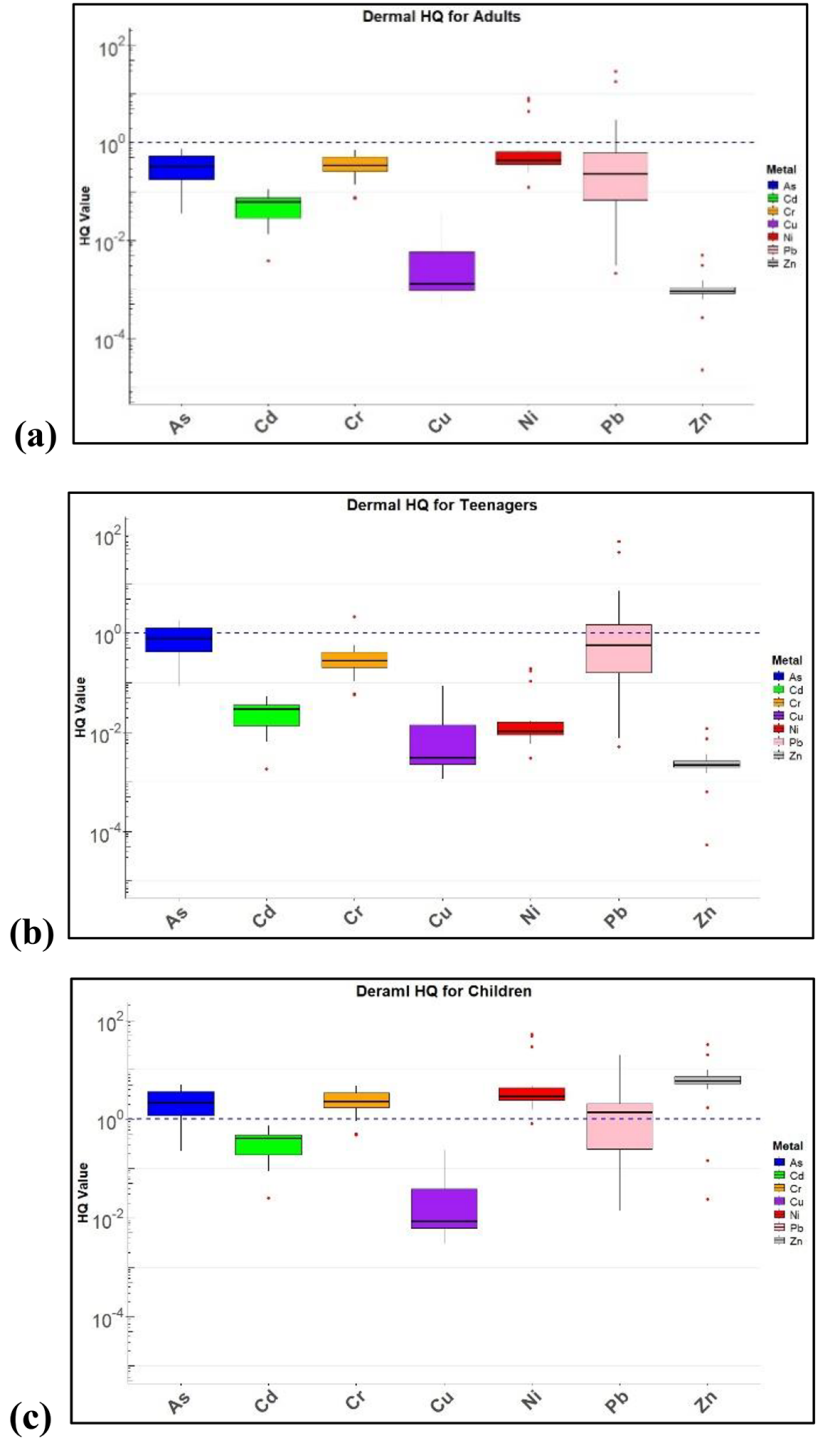
HQ values for dermal contact in (**a**) Adults, (**b**) Teenagers and (**c**) Children.

Non-CR health risks were assessed for As, Zn, Cr, Pb, Ni, Cu, and Cd through dermal contact and oral ingestion in adults, teenagers, and children. HQ values in all age groups from oral and dermal exposure are shown in Figs. 6 and 7.

**Fig. 8:**
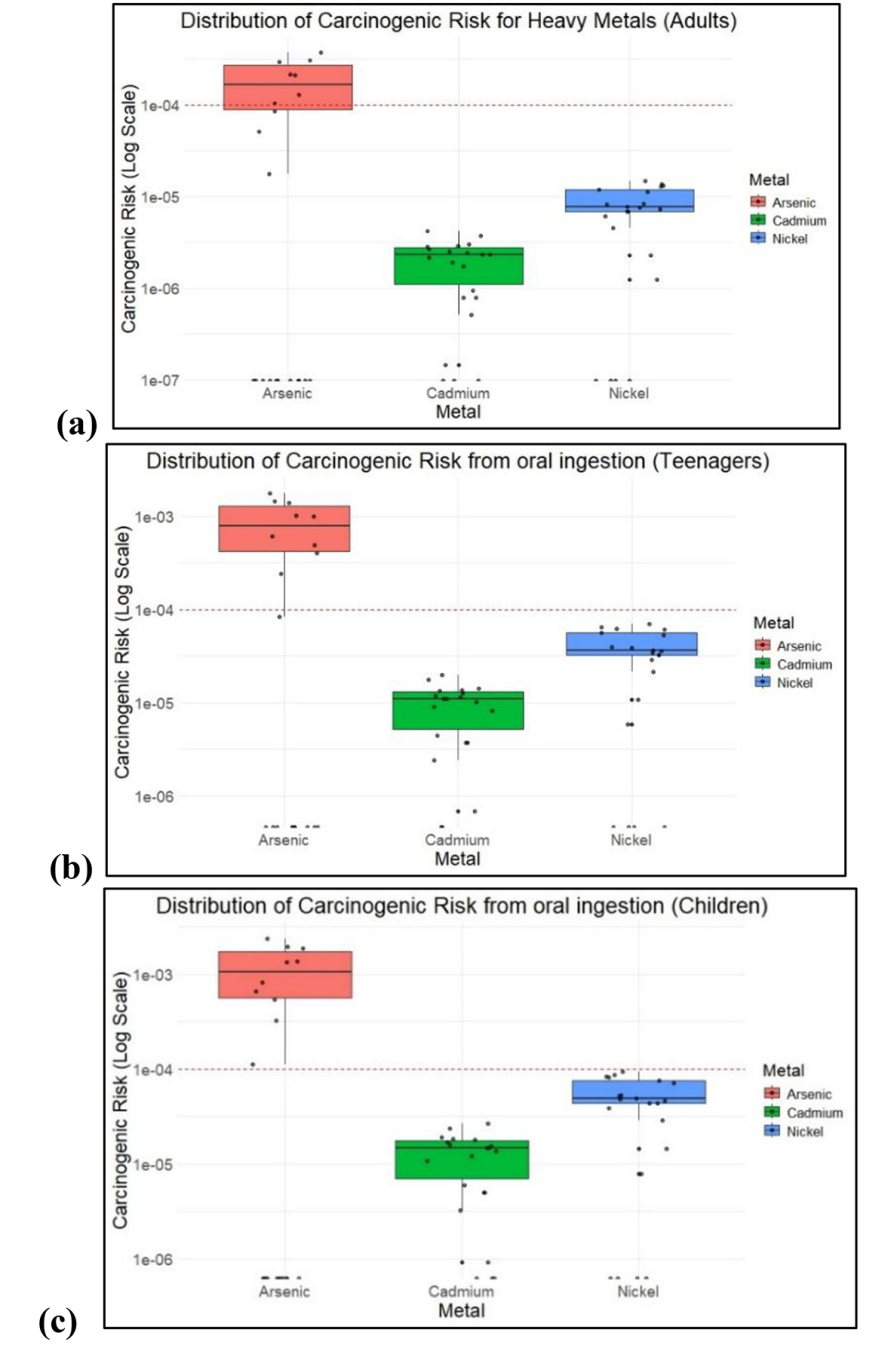
Carcinogenic risk values across samples via oral ingestion in (**a**) Adults, (**b**) Teenagers, and (**c**) Children.

**Fig. 9:**
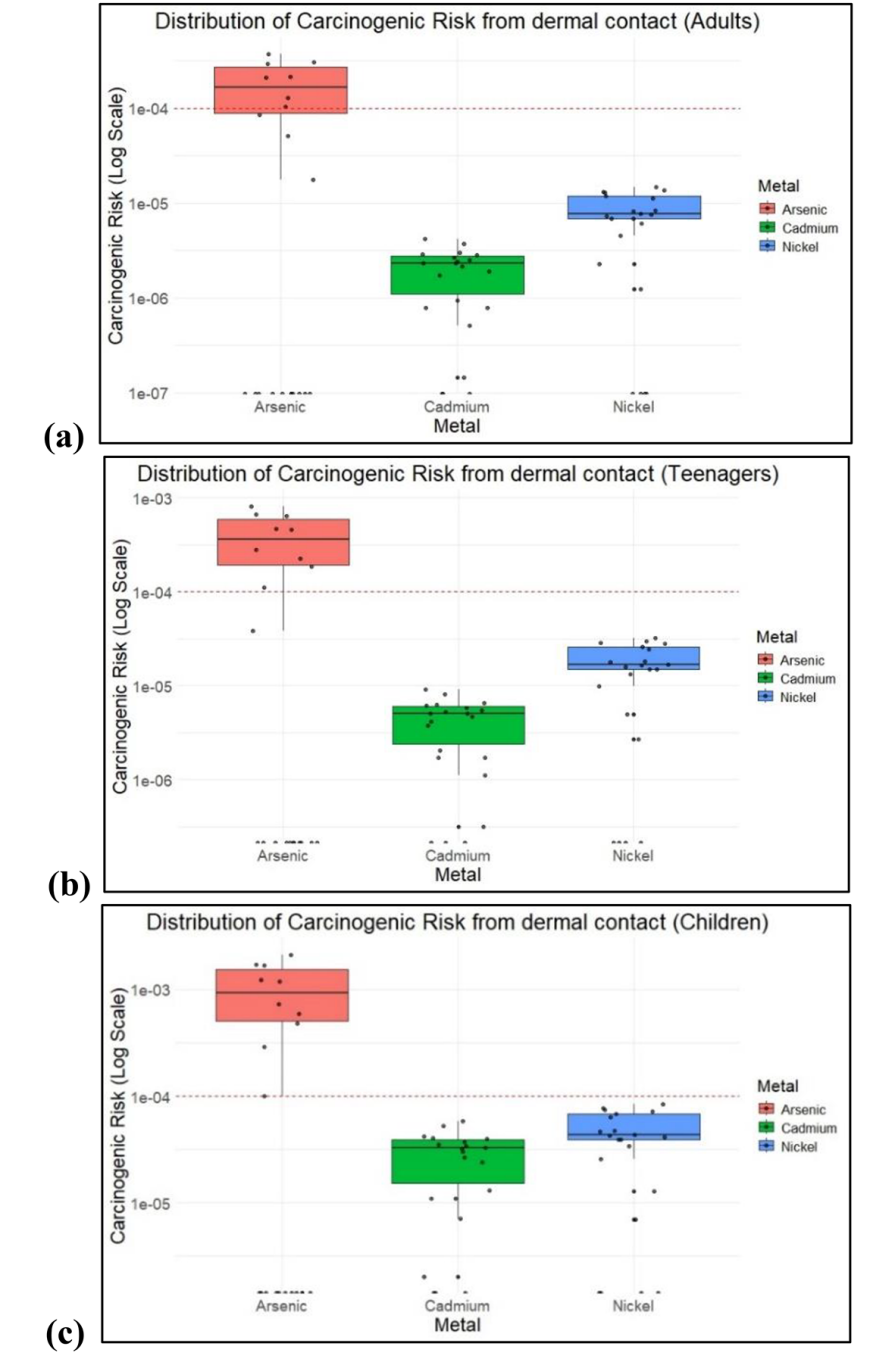
Carcinogenic risk values across samples via dermal contact in (**a**) Adults, (**b**) Teenagers, and (**c**) Children.

Among these, 34% of the samples surpassed the safe value of 1E10-4 for adults and teenagers, whereas 47% of the samples surpassed the safe value for children via oral ingestion of As. The CR values of Cd and Ni were well within the limits, indicating lower carcinogenic risk. Whereas CR values of As from dermal contact varied from 1E-5 to 3.72E-4, 1E-5 to 8.05E-4, and 2.1E-3 in adults, teenagers, and children respectively. The CR values for Cd and Ni were within 1E-4. CR values of As, Ni, and Cd were well within carcinogenic risk from inhalation. Table 5 shows the distribution of CR values in all age groups after oral ingestion, dermal contact and inhalation. Figures 8 and 9 shows the distribution of CR values in all age groups.

**Table 5:** CR values of heavy metals from oral ingestion, dermal contact and inhalation in all age groups (values less than 10^− 10^ are very low risk values. Therefore, such values are given as zero).

Metals	Exposure pathway	Adults		Teenagers		Children	
		Max	Average	Max	Average	Max	Average
**Arsenic**	Oral	8.40E-04	1.92E-04	0.09E − 4	0.04E − 04	0.60E − 04	0.27E-04
	Dermal	3.72E-04	0.08E − 04	8.05E-04	1.84E-04	21.0E − 04	4.81E-04
	Inhalation	0.0005E − 04	0.0001E-4	0.0001E-4	0.00003E-4	0.01E-4	0.0002E-4
**Cadmium**	Oral	17.6E-4	4.03E-04	0.19E-4	0.08E-4	0.706E-4	0.32E-4
	Dermal	0.04E-4	0.01E-4	0.09E-4	0.03E-4	0.58E-4	0.25E-4
	Inhalation	0	0	0	0	0	0
**Nickel**	Oral	23.5E-4	5.37E-04	0.26E-4	0.11E-4	0.94E-4	0.43E-4
	Dermal	0.49E-05	0.68E-04	3.22E-04	0.14E-05	0.84E-05	0.39E-05
	Inhalation	0	0	0	0	0	0

Figures 8 and 9 illustrate the carcinogenic risks from oral ingestion, dermal contact of As, Cd, and Ni in adults, teenagers, and children.

### Monte Carlo simulation

To anticipate a set of CR values for oral ingestion and dermal absorption of As, Cd, and Ni, a Monte Carlo simulation was run between the real-time data. CR values were simulated for As, Cd, and Ni after oral ingestion and dermal absorption. These values were evaluated in adults, teenagers, and children.

**Fig. 10:**
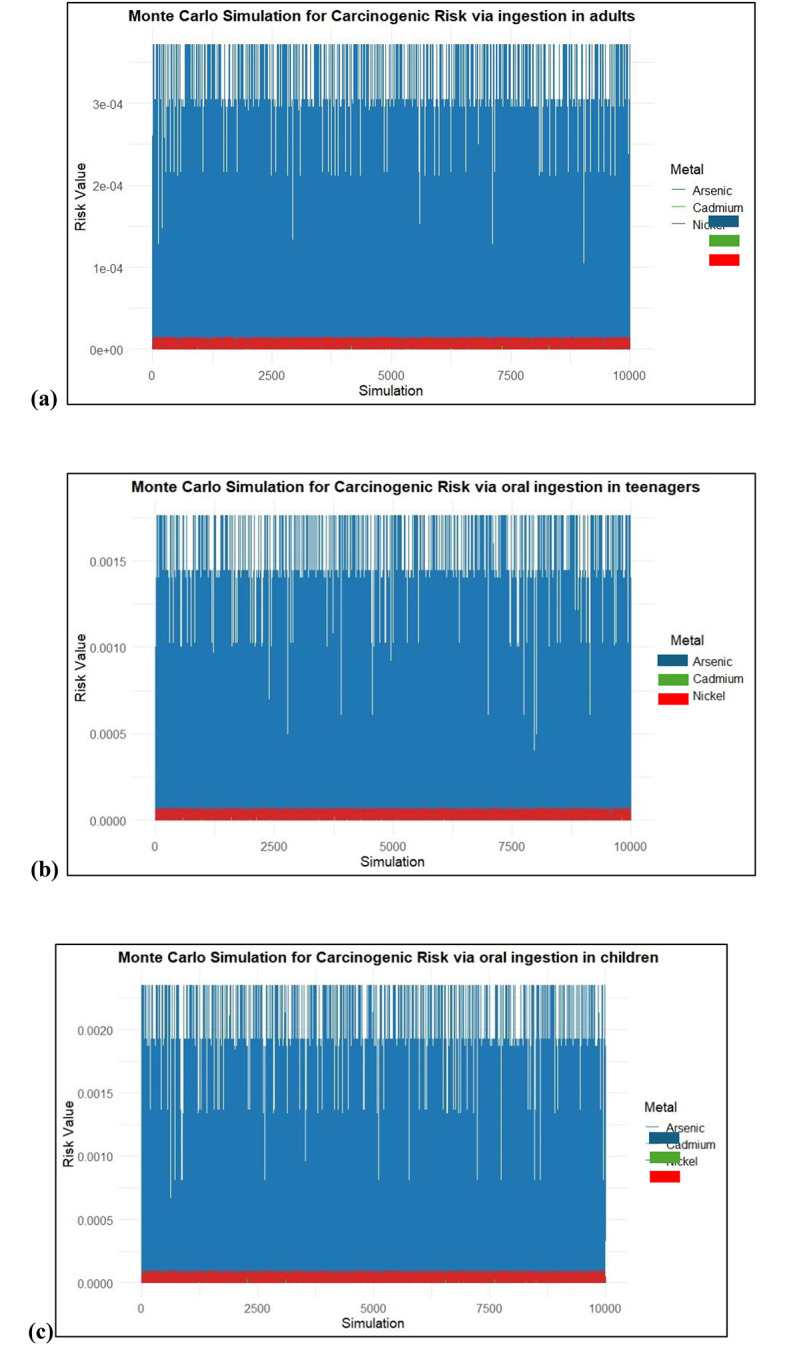
CR values for oral ingestion of heavy metals (**a**) Adults, (**b**) Teenagers and (**c**) children.

Figure 10 shows that As posed a high risk with consistently higher risk values across all simulations. The risk values for As fluctuated but remained mostly within the range of 1 × 10 − 4 to 3E10 − 4 indicating that the simulations consistently predicted a significant carcinogenic risk from As ingestion in adults; however, the simulations for risk in teenagers and children were significantly higher. It was observed that the As risk value ranged from 2E10-3 in children, indicating a higher risk and requiring attention to monitor the soil samples in the region. Cd and Ni showed a lower risk compared to As, with a range of 1E10-5. The calculated mean CR values for adults are 4.87E-04 for As, 7.58E-06 for Cd, and 3.25E-05 for Ni, with simulations indicating 0.00 at the 5th percentile and 8.40E-04, 6.07E-05, and 9.41E-05 at the 95th percentile for As, Cd, and Ni, respectively. For teenagers, the calculated mean CR values are 6.88E-04 for As, 1.92E-05 for Cd, and 3.75E-05 for Ni, with simulated 5th percentile values of 0.00 and 95th percentile values of 1.76E-03 for As, 3.63E-05 for Cd, and 5.67E-05 for Nickel. In children, the calculated mean CR values are 8.22E-04 for As, 2.04E-05 for Cd, and 4.79E-05 for Ni, with simulation values showing 0.00 at the 5th percentile and 2.35E-03, 7.56E-05, and 8.37E-05 at the 95th percentile for As, Cd, and Ni respectively.

At the 95th percentile, the carcinogenic risk (CR) from As reaches its highest potential, with values likely around 3E10 − 4 for adults, 1.5E10-3 for teenagers, and 2E10-3 for children. This suggests that, under worst-case scenarios, a significant portion of the adult population could experience elevated cancer risks due to As exposure through ingestion, but teenagers and children are at a high risk of As contamination. Ni and Cd are present at much lower risks at this percentile level, with Ni barely registering any substantial risk.

**Fig. 11:**
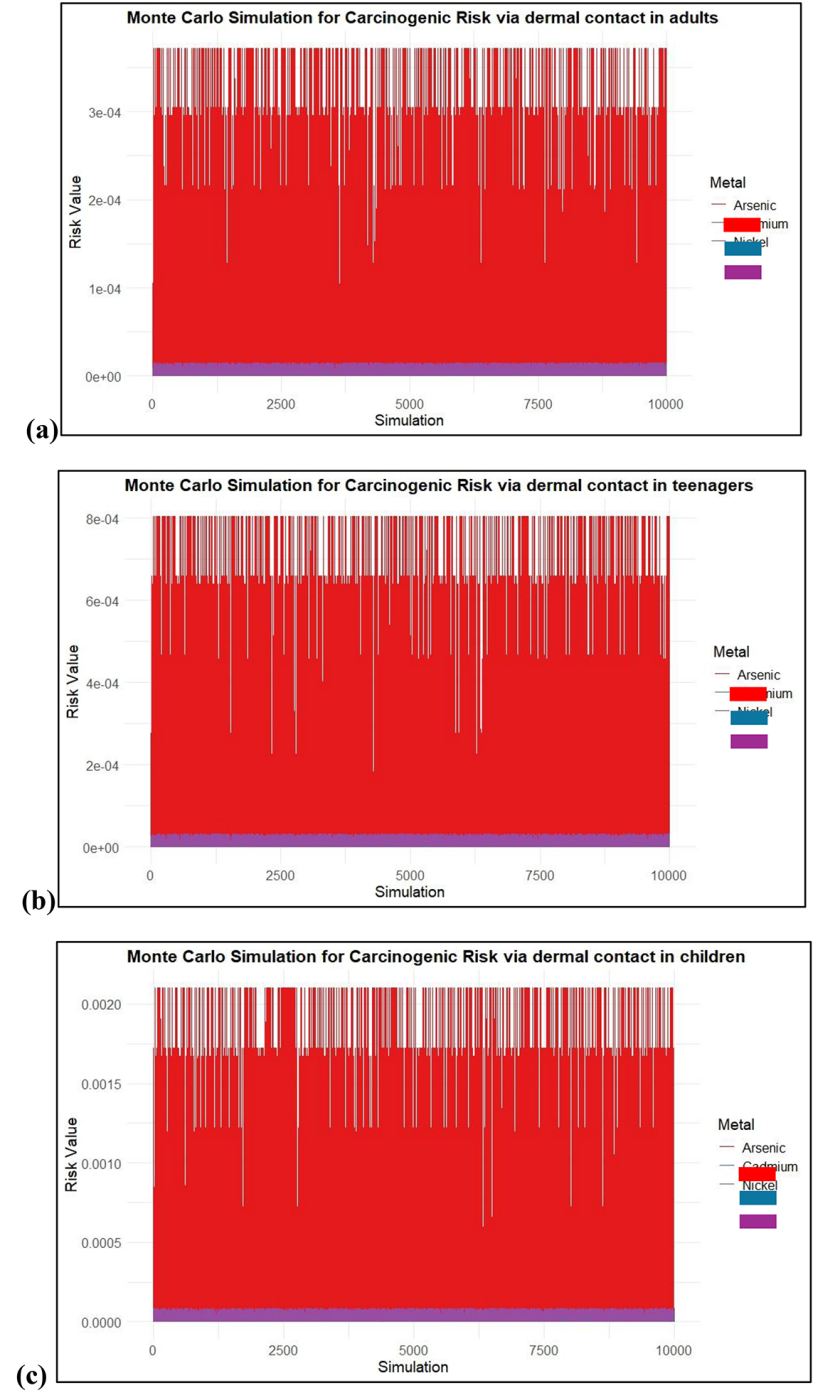
CR values for dermal contact of heavy metals (**a**) Adults, (**b**) Teenagers and (**c**) children.

Figure 11 illustrates the results of a Monte Carlo simulation assessing the carcinogenic risks of As, Cd, and Ni across different age groups via dermal contact. The calculated mean CR values for dermal exposure indicate that adults have an As mean of 3.22E-04, with simulation results showing a minimum of 0.00E + 00 at the 5th percentile and reaching 3.71E-04 at the 95th percentile. Teenagers show a mean CR for As at 5.42E-04, with simulation results ranging from 0.00E + 00 to 6.71E-04. For children, the calculated mean CR for As is 1.28E-04, and the Monte Carlo simulations reflect a range from 0.00E + 00 to 1.22E-04 at 95th percentile. These findings underscore the potential risks associated with dermal exposure across different age groups, particularly for As.

**Fig. 12:**
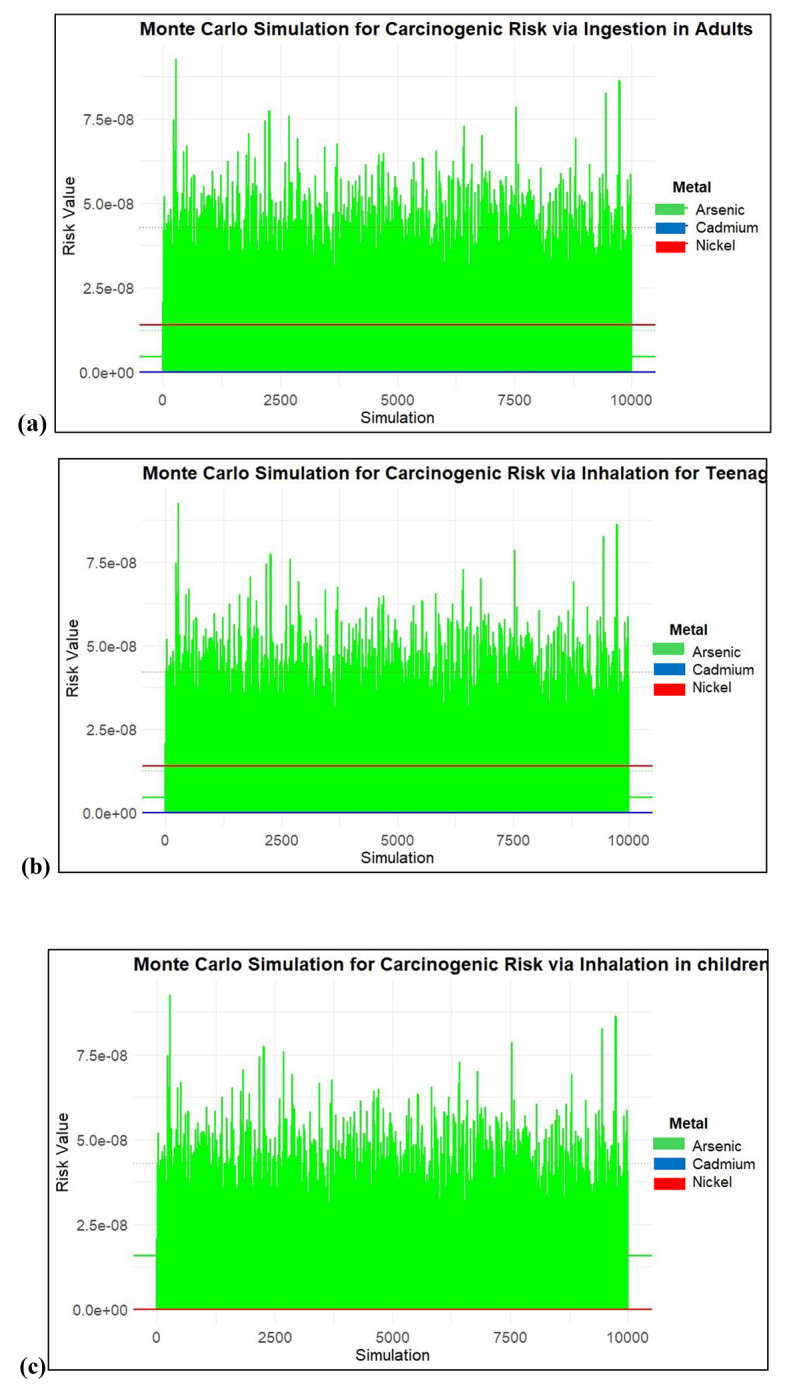
CR values for inhalation of heavy metals (**a**) Adults, (**b**) Teenagers and (**c**) children.

Figure 12 shows the differences between calculated and simulation simulation results across adults, teenagers, and children. For adults, the calculated mean for Arsenic is 1.61E-08, while the Monte Carlo 5th percentile shows no risk (0.00E + 00), and the 95th percentile indicates a higher potential risk at 5.54E-08. Similarly, for teenagers, the calculated mean for Arsenic is notably lower at 1.16E-09, with the Monte Carlo 5th percentile remaining at zero and the 95th percentile rising to 1.3E-08. Children exhibit a higher calculated mean for Arsenic at 3.92E-08, with the Monte Carlo simulations again revealing no risk at the 5th percentile and a significant increase to 1.2E-07 at the 95th percentile.

## Discussion

### Sources and availability of heavy metals in soil

The spatial patterns observed in these maps highlight a combination of anthropogenic and natural sources that contribute to heavy metal contamination in the study area.

**Fig. 13:**
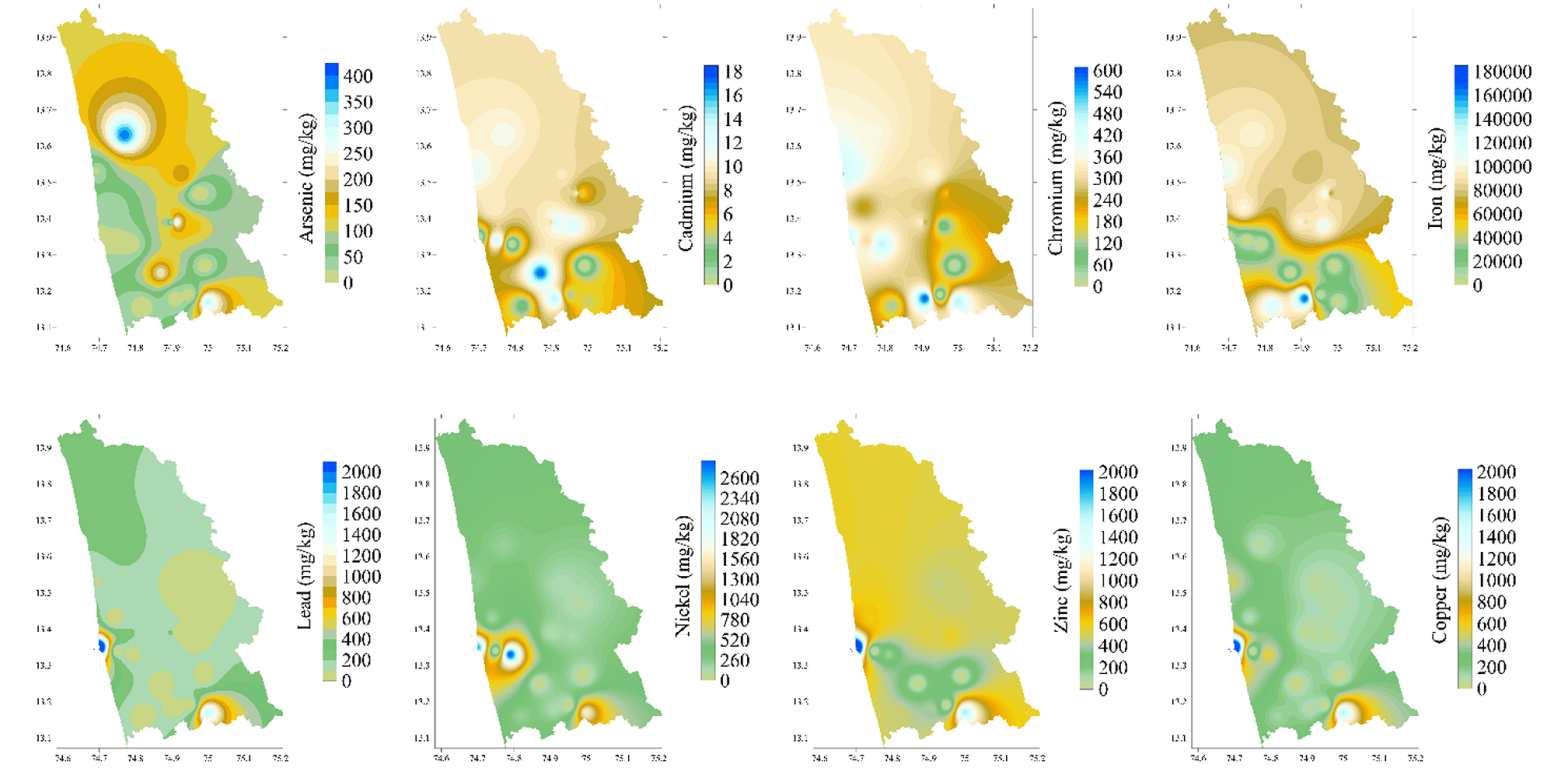
Spatial distribution As, Cd, Cr, Fe, Pb, Ni, Zn, and Cu in soil samples collected from the Udupi district.

Figure 13 shows the spatial variation of As concentrations, with values ranging from 0.007 mg/kg to a maximum of 391.822 mg/kg. Higher values are observed in the northern and central parts of the area, particularly around industrial zones such as chemical plants and clay tile industries (S8, S9, S13). These areas significantly exceed WHO’s recommended limit of 10 mg/kg, indicating heavy anthropogenic influence. The southern region displays relatively lower arsenic concentrations. The localized distribution of As is often due to geological factors such as the presence of arsenic-rich minerals and geothermal activity, which release As into the environment^51^. Highest concentration of Cd was observed to be 17.45 mg/kg, showing a uniform distribution throughout the study area. The spatial distribution of cadmium (Cd), as shown in the map, reveals a concentration range from 0.001 mg/kg to 17.45 mg/kg. The higher concentrations are notably observed in southern industrial areas, particularly around the fish mills and coal power plant regions. The presence of elevated Cd levels in these areas points to significant anthropogenic influence, primarily due to industrial activities. The concentration exceeds both national guidelines and international standards set by the ISO, which typically range from 0.8 to 3 mg/kg, and the WHO. The spatial distribution of Cr concentrations across Udupi District, as shown in the figure, indicates significant variability, with values ranging from 0.03 mg/kg to 589.209 mg/kg. The southern and central regions, especially areas around S5, S18, and S19, show higher concentrations, suggesting localized contamination likely influenced by industrial activities such as metal processing and waste discharge. The northern parts exhibit comparatively lower levels of Cr, pointing towards limited anthropogenic influence. While some natural sources, such as the weathering of Cr-bearing minerals, may contribute to background levels, the elevated concentrations are primarily linked to industrial output. The observed concentrations exceed both the Indian Standard limit of 47 mg/kg for Cr in soils and the WHO guideline of 100 mg/kg. Spatial distribution of Fe varied from 6.574 mg/kg to 166,446.62 mg/kg, shows elevated levels in the southern region. The high Fe concentrations may be attributed to both anthropogenic and natural factors. Industrial activities, such as metal processing and foundries, can contribute to localized Fe contamination (S5, S6, S18, S19). Naturally, the presence of iron-rich laterite soils, typical in tropical regions like Udupi, may also explain the elevated background levels (in S8 and S16). The area’s geomorphology and natural weathering of iron-bearing minerals in the soil further contribute to this distribution. Concentration of Ni varied from 0.047 mg/kg to 2608.15 mg/kg in soil. The mean value was observed to be 2832.579 mg/kg. Ni was higher in the southern region of the study area. This localized enrichment is indicative of potential anthropogenic sources, likely due to the industrial activities in this area. The central and northern parts of the region exhibited relatively low concentrations, suggesting low contamination. From the spatial distribution of Pb shows concentrations of Pb varied from 0.222 mg/kg to 2294.235 mg/kg. Elevated levels were observed in the southern part of the study area, notably around industrial zones such as foundries, rice mills, and chemical industries (S5, S6, S18, S19). This result is consistence with a previous study conducted in the region indicating anthropogenic contamination of Pb^5^. However, natural sources, such as the weathering of lead-containing rocks, may also contribute to the background levels observed in less industrialized areas. Despite this, anthropogenic sources, particularly industrial emissions, appear to be the primary driver of contamination, far exceeding WHO (100 mg/kg) and Indian soil standards. The spatial distribution of Ni concentrations reveals significant variability, with prominent concentrations near industrial zones such as fish mills, rice industries, and a coal power plant (notably S5, S6, S18, S19). The highest recorded value of Ni (2608.15 mg/kg) far exceeds both WHO guidelines (75 mg/kg) and Indian soil standards (100 mg/kg). These findings indicate severe contamination, particularly in industrial areas, suggesting substantial anthropogenic influence on soil quality. Zn concentrations ranged from 0.033 to 2,625 mg/kg. The Zn levels varied significantly, with the majority of the regions displaying lower concentrations. Elevated concentration of Zn can be attributed industrial activities and extensive agricultural practices^52^. The natural background value of Fe in the region was found to be ranging to 300 mg/kg. Concentration Cu varied from 0.019 mg/kg to 2499.56 mg/kg. It shows contamination hotspots in the southern region, particularly near sampling sites S5 and S6. These areas are close to industrial activities, such as fish processing units and thermal power plants, which likely contribute to the elevated copper levels. However, the overall distribution pattern indicates a higher influence of natural sources^53^. The observed concentrations exceed WHO limit of 100 mg/kg. The natural sources of Cu, Fe and Zn, in Udupi soils can be attributed to the region’s geology and weathering processes. Udupi’s soils are rich in laterites, which form through intense weathering of basaltic and granitic rocks. These rocks naturally contain copper-bearing minerals like chalcopyrite, bornite, and malachite. The tropical climate, high rainfall, and the presence of rich mineral deposits from the Western Ghats further enhance the leaching of these metals into the soil, contributing to their natural concentrations in the region.

### Environmental risk assessment

The CF and Igeo values in the study area indicate significant pollution from both industrial and natural sources. As shows high CF (19.59) and Igeo (5.71) values, with the thermal power plant (S20) likely being the main contributor due to emissions from power generation processes. Cd contamination, with CF values reaching 6.45 and Igeo values peaking at 5.43, is mainly associated with chemical industries (S2, 4, 10, 19) and paint and enamel industries (S1), where Cd is used in pigments and industrial processes. Cr, with CF values up to 12.55 and Igeo values up to 2.48, can bedue to chemical industries, foundries (S17), and clay tile industries (S7, 9), but may also have a natural contribution from the weathering of chromium-rich minerals. Cu, with CF values up to 18.52 and Igeo up to 6.38, is likely from oil and fish meal industries (S3, 5, 6, 21), as well as foundries and chemical industries, though natural sources such as copper-bearing minerals could contribute in certain areas. Ni shows extreme contamination, with CF up to 37.77 and Igeo up to 5.98, primarily linked to the thermal power plant, chemical industries, and oil industries (S3). Some of the Ni pollution could also be attributed to natural sources like ultramafic rocks. Pb contamination (CF up to 10.36 and Igeo up to 6.43) is associated with paint and enamel industries, chemical industries, and foundries, where lead-based compounds are used. Zn (CF up to 8.75 and Igeo up to 5.13) is linked to oil and fish industries, chemical industries, and fish nets industries (S8), but zinc can also be released naturally from soil erosion and weathering of zinc-containing rocks. Iron (Fe) contamination remains low but could originate from foundries, clay tile industries, and natural soil mineralogy^54,55^. PLI values across various locations indicate significant variability in contamination levels. The highest PLI values are observed in a few samples, with values reaching up to 5.66, indicating severe pollution. These high contamination levels necessitate immediate attention for pollution mitigation and risk assessment due to the possible health risks related to such high levels of pollution. Several other samples show moderate PLI values, ranging from 1.37 to 2.69. These areas, while not as critically polluted as the highest contamination zones, still require careful monitoring and management to prevent escalation of pollution levels. A few samples exhibit very low PLI values, some as low as 0.01, indicating minimal pollution. These low-contamination areas are currently at low risk, but continuous monitoring is essential to ensure that pollution levels remain controlled. Overall, the data underscores the necessity for targeted environmental management strategies to address areas with high contamination, while maintaining vigilance in less affected regions to safeguard public health and the environment.

### Non-carcinogenic health risk

Non-carcinogenic risk assessment showed that, for As, the HQ values across both oral and dermal routes consistently indicated high risk, particularly in children. The oral HQ values for As were significantly elevated, with the children’s median values exceeding the critical threshold of 1, indicating substantial risk. Similarly, in the dermal HQ plots, the children showed the highest median values, underscoring their heightened vulnerability to As exposure through both ingestion and skin contact. Cd presented a similar pattern in the oral exposure scenario, where children demonstrated considerably higher HQ values than adults and teenagers, indicating greater susceptibility. While the HQ values for Cd from the dermal intake were lower, children still exhibited higher median values compared to other age groups, indicating that dermal exposure poses a non-negligible risk, particularly for younger individuals. Cr and Cu exhibited lower HQ values, especially with dermal exposure, where Cu showed a relatively low risk across all age groups. However, oral exposure to Cr presents a more varied risk, with teenagers showing wider distributions, suggesting variability in the exposure or absorption rates. Ni and Zn both showed lower HQ values overall, with Ni exhibiting slightly higher dermal HQ values in teenagers, potentially due to lifestyle or environmental factors unique to this group. Zinc showed low HQ values across both exposure routes, indicating minimal risk in this context. Pb is a major concern, especially in children. The HQ values for Pb in both the oral and dermal exposure scenarios were elevated, with many exceeding the critical threshold, particularly in oral exposure. This highlights the significant risk that Pb poses to children, who are known to be more susceptible to its toxic effects, particularly regarding neurological development. Non-carcinogenic risk from inhalation were significantly low and negligible. In this study area, non-carcinogenic risk from dermal intake of heavy metals in children were greater than other age groups which is consistent with other studies as shown in Table 6. However, few studies show higher carcinogenic risk compared to non-carcinogenic risk. Children are at higher risk due to their behavioural patterns such as they are highly likely to engage in hand- to- mouth behaviour and play habits. As their systems are still developing, it makes then very sensitive to the toxicity of metals. The findings of this study reveal that children have a heightened vulnerability to the cognitive and neurological risks posed by lead (Pb), cadmium (Cd), and arsenic (As).

### Carcinogenic health risk

Carcinogenic risk data revealed that As poses the most significant risk, with adults, teenagers, and children showing median risk levels that exceed the general safety threshold limit. Carcinogenic risk values indicate that children are subjected to higher risk, followed by teenagers, suggesting greater vulnerability, possibly due to differences in metabolism, body weight, or exposure patterns. As is one of the most studied carcinogens and the significant CR values of As is consistent. However, Cd showed a lower and more consistent risk across all age groups, although the distribution for children was broader, indicating more variability in risk. Ni, while presenting a moderate risk, exhibits similar patterns among all age groups but with a wider distribution in children and teenagers, showing varied exposure or biological responses. This evaluation emphasizes carcinogenic risks in teenagers, particularly from oral ingestion and dermal contact.

**Table 6:** Environmental and Health risk assessment across studies.

Metals analysed	Findings	Reference
As, Cr, and Ni	10^− 4^> CR > 10^−^5; CR_(Children)_ > CR_(Adults);_ HI > 1	^56^
As, Cr, Cu, Zn, Ni, and Pb	I_geo_ indicated moderate to extreme pollution; CR for children and adults from As and Cr; HI < 1	^57^
Pb, Cr, Cu, Zn, Co and Ni	I_geo_ indicated moderate Pb and Co pollution; EF shows minor to severe anthropogenic pollution; HI_(Children)_ > HI_(Adults);_ CR from Cr; Non-CR from Cr and Pb	^58^
Cr, Cu, Zn, Pb, Cd, V and As	Non-CR from heavy metals; CR from As in both adults and children; Cd, Zn, As, and Pb from high temperature coal combustion.	^59^
Cu, Pb, Cr, Zn and Fe	No carcinogenic risk; HI_(Children)_ > HI_(Adults)_; Non-CR from Pb	^60^
Co, Pb, Co, and Pb	EF indicated anthropogenic pollution; No significant Non-CR and CR	^61^
Pb, Cr, As, Cu, Cd, and Ni	CR > Non-CR; Non-CR from Cd; CR_(Dermal)_ > CR_(Inhalation and Ingestion)_ in children	^62^
Ag, Al, As, B, Ba, Ca, Cd, Co, Cr, Cu, Fe, Hg, K, Li, Mg, Mn, Na, Ni, Pb, Sr, and Zn	HQ _(Dermal)_ > HQ _(Ingestion)_ > HQ _(Inhalation)_; Ag, Cd, Cr, Cu, Co, Ni, and Pb posing ecological risks; Fe and Cr show high non-CR risks; Cr and Ni show high CR	^63^
Cu, Pb, Zn, Ni, Cr, Fe, Mn, Cd, As and Se	Non-carcinogenic risk from Mn; No CR from As; HQ_(Dermal)_ > HQ_(Ingestion and Inhalation)_	^64^

The Monte Carlo simulation for CR results indicates that even at the lower bound of risk estimates, children face the highest potential risk from arsenic exposure through skin contact. The simulation further revealed that the 95th percentile CR values for arsenic in children and teenagers were 2.3E10-3 and 8E10-4 respectively. In contrast, the estimated CR values for adults in the 95th percentile were less than 4E10-4. The results show that children and teenagers have a significantly increased chance of developing cancer from dermal exposure to As, even though the risk is still significant for all age groups. In all age categories, the calculated CR values for Cd and Ni were less than 1E10-4, suggesting a lower risk of dermal exposure. For As, the estimated CR values exceeded the acceptable risk level of 1E10-4. This emphasizes the urgent need to minimize As contamination in soil and develop effective mitigation and removal strategies to protect vulnerable populations, especially children and teenagers, from the heightened carcinogenic risks associated with arsenic exposure through dermal contact.

## Conclusion

This study evaluated contamination level and health risk associated with heavy metals in soil samples of selected study area. ICP-OES was used to assess elemental concentration in soil samples. In collected soil samples, the data obtained was considered for geospatial analysis, indices and Monte Carlo simulation. The results indicated non-CR and CR in the population exposed to contaminated soil. The study population subgroups were adults, above age 11, below age 11. Risk indices such as Igeo, CF, PLI, HQ, HI, and CR were used. Results indicated that over 60% of soil samples were contaminated (CF > 6, Igeo > 5, and PLI > 1) along with non-carcinogenic health risks (HQ > 1) observed in most samples which can be corelated to health risk posed to below age 11. Hazard Index values exceeded 1 across all age groups exposed to contamination. CR values for metalloid in particular, suggested high carcinogenic risk for 33% of the population of study area.

Monte Carlo simulations enhance precision which is helpful in environmental risk assessment. These simulations highlighted the variability and uncertainty in risk assessments, revealing higher risks for below age 11 and confirming carcinogenic effects for metals like As and Pb in soil samples. Although CR values for Cd and Ni were below critical thresholds, the need for remediation is emphasized due to their toxicity at low concentration. This study suggests the importance of targeted intervention, particularly susceptible populations below age 11 exposed to contaminated soil.

### Recommendations for Future Research and Policy Changes

- Soil remediation protocols must be enforced, particularly in areas with elevated levels of As, Pb, and Cd, is essential. Regulatory agencies must continuously monitor remediation protocols near industrial areas to reduce HM contamination in soil.
- Since the results show higher risk to children especially via dermal contact of soil, regulatory measures need to be implemented to avoid direct contact with contaminated soil.
- Future studies can conduct long-term epidemiological and seasonal studies to monitor health outcomes of populations that are exposed to HM.
- Heavy metal speciation and bioavailability studies in this region can address exact source of contamination and capacity of intake of these HM to food products.

## Electronic supplementary material

Below is the link to the electronic supplementary material.

Supplementary Material 1

## Data Availability

The datasets generated and/or analysed during the current study are not publicly available as it is not published elsewhere but are available from the corresponding author on reasonable request.
