# Supplementary material for: Assessment of Carcinogenic and non-carcinogenic risk indices of heavy metal exposure in different age groups using Monte Carlo Simulation Approach
Source: Sci Rep. 2024 Dec 5;14:30319. doi: 10.1038/s41598-024-81109-3 (PMC11621557; doi:10.1038/s41598-024-81109-3)
Supplement: Supplementary file 1 — Supplementary Material 1 [file 41598_2024_81109_MOESM1_ESM.docx]

**Assessment of Carcinogenic and Non-Carcinogenic Risk Indices of Heavy Metal Exposure in Different Age Groups using Monte Carlo Simulation Approach**

**B Raksha Shetty^1^, Jagadeesha Pai B^1*^, Salmataj S A^2*^, Nithesh Naik^3^**

^1^Department of Civil Engineering, Manipal Institute of Technology, Manipal, 576104

^2^Department of Biotechnology, Manipal Institute of Technology, Manipal, 576104

^3^Department of Mechanical and Industrial Engineering, Manipal Institute of Technology, Manipal, 576104

*Corresponding author

***APPENDIX1: Figures***


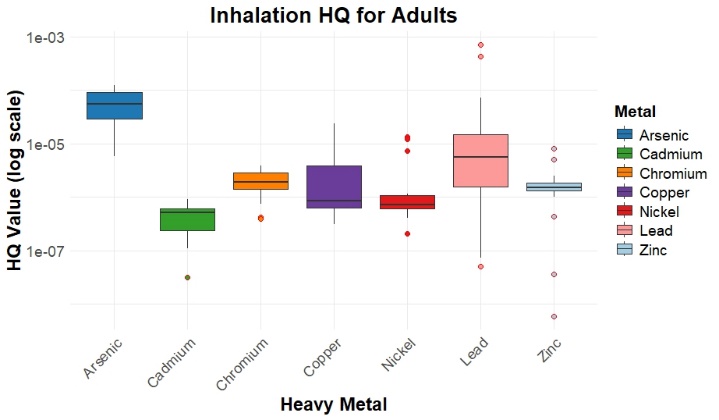


Figure 1: HQ values for inhalation in adults


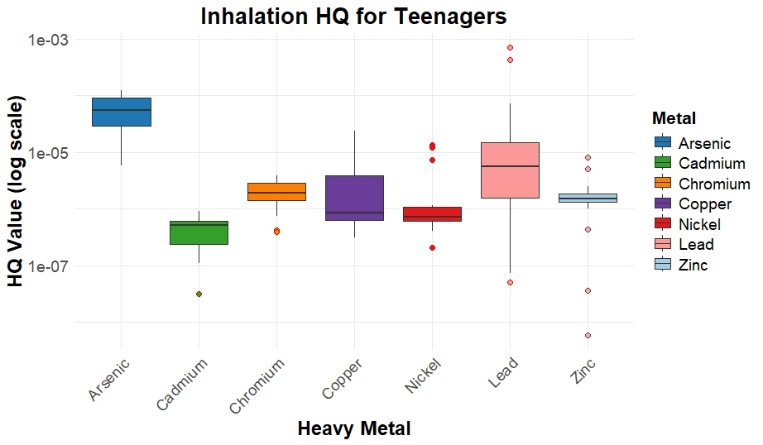


Figure 2: HQ values for inhalation in teenagers


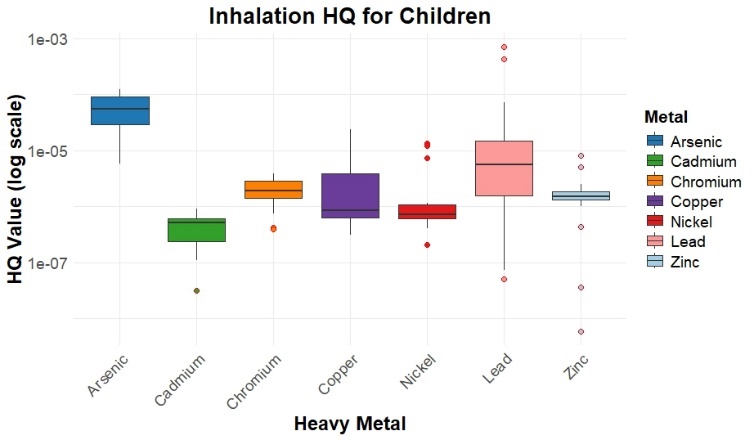


Figure 3: HQ values for inhalation in Children


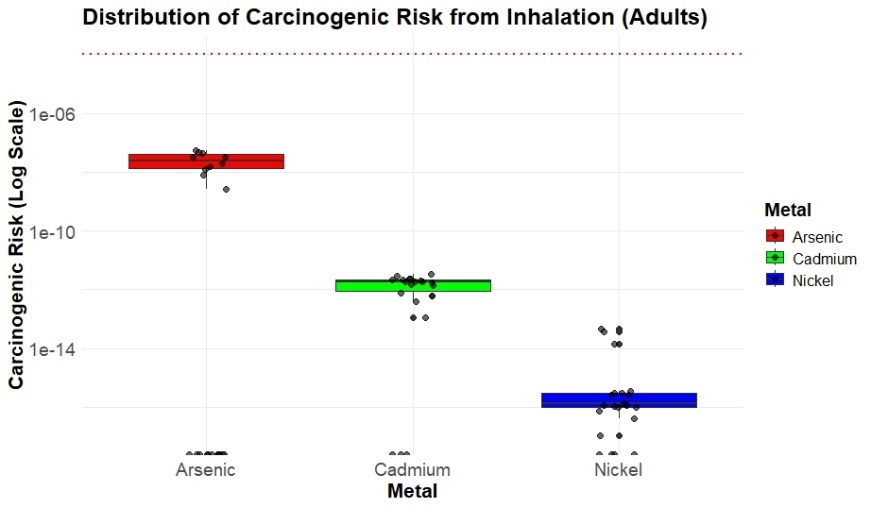


Figure 4: Carcinogenic risk values across samples via inhalation in adults


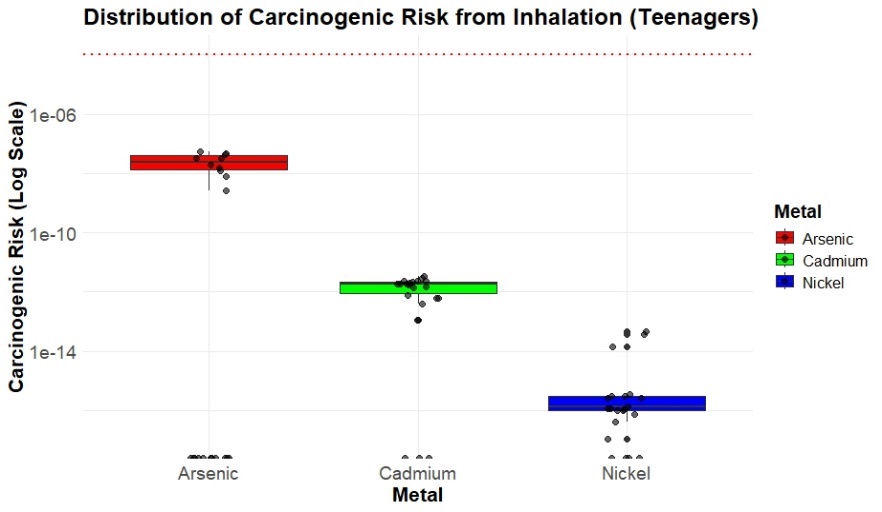


Figure 5: Carcinogenic risk values across samples via inhalation in teenagers


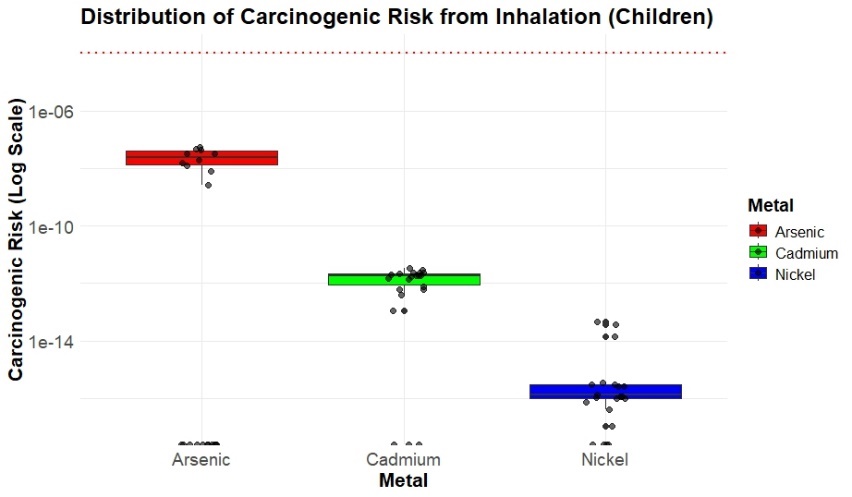


Figure 6: Carcinogenic risk values across samples via inhalation in children
